# The effect of high concentration of zoledronic acid on tooth induced movement and its repercussion on root, periodontal ligament and alveolar bone tissues in rats

**DOI:** 10.1038/s41598-021-87375-9

**Published:** 2021-04-07

**Authors:** Fátima Regina Nunes de Sousa, Vanessa Costa de Sousa Ferreira, Conceição da Silva Martins, Hugo Victor Dantas, Frederico Barbosa de Sousa, Virgínia Cláudia Carneiro Girão-Carmona, Paula Goes, Gerly Anne de Castro Brito, Renata Ferreira de Carvalho Leitão

**Affiliations:** 1 Post-Graduation Program in Morfofuncional Sciences (PCMF), Departamento de Morfologia, Faculdade de Medicina, Medical School, Universidade Federal do Ceará (UFC), Rua Delmiro de Farias, s/n, Rodolfo Teófilo, Fortaleza, CE 60441-750 Brazil; 2 Department of Pathology and Legal Medicine, Medical School, Federal University of Ceará (UFC), Rua Monsenhor Furtado, s/n, Fortaleza, CE 60441-750 Brazil; 3 Department of Morphology, Medical School, Federal University of Piauí (UFPI), Rua Cícero Duarte, 905, Picos, PI 64607-670 Brazil; 4 Graduate Program in Dentistry, Health Sciences Center, Federal University of Paraíba (UFPB), Campus I, Cidade Universitária, João Pessoa, PB 58059-900 Brazil

**Keywords:** Health care, Risk factors

## Abstract

Zoledronic acid (ZA) is often prescribed for osteoporosis or resorptive metabolic bone disease. This study aims to evaluate the effect of ZA on orthodontic tooth movement (OTM) and root and bone resorption and its repercussion on root, periodontal ligament and alveolar bone tissues. The experimental group consisted of 72 Wistar rats divided in four subgroups: Naive, Saline and Zoledronic Acid groups at the concentration of 0.2 mg/kg [ZA (0.2)] or 1.0 mg/kg [ZA (1.0)]. The animals were subjected to i.v (dorsal penile vein) administrations of ZA or saline solution, on days 0, 7, 14 and 42. Under anesthesia, NiTi springs were installed in the first left maxillary molar with 50gf allowing the OTM, except for the negative control group (N) for mesial movement of the left first maxillary teeth. The animals were sacrificed and maxillae were removed for macroscopic and histopathological analyzes, scanning electron microscopy, computerized microtomography and confocal microscopy. Treatment with ZA decreased the OTM and the number of osteoclasts and loss of alveolar bone when compared to the naive and saline groups. Reduction of radicular resorption, increased necrotic areas and reduced vascularization in the periodontal ligament were observed in the ZA groups. ZA interferes with OTM and presents anti-resorptive effects on bone and dental tissues associated with a decreased vascularization, without osteonecrosis.

## Introduction

Bone tissue is a special type of connective tissue, rigid, highly vascularized and metabolically active^1–3^. Bone metabolism is regulated by osteoclasts, the only bone resorbing cells^4^, osteoblasts, cells engaged in the synthesis, glycosylation and secretion of proteins^5^ and osteocytes, residing within the bone matrix. Accumulating evidence supports that osteoblasts and osteocytes play an important role in osteoclastogenesis and bone resorption^6^.

Bone remodeling allows tooth movement through orthodontic devices, which when applying force to the center of the tooth, results in the distribution of mechanical stress to the periodontium, promoting bone resorption on the compression side and bone neoformation on the tension side^7^. The biology of tooth movement is modified by a series of conditions, such as strength intensity, type of movement, observation period, teeth involved, root and bone crest morphology and the influence of drugs^8,9^.

Different drugs, such as non-steroidal and steroidal anti-inflammatory, simvastatin, atorvastatin, strontium ranelate^10^ and more recently bisphosphonates (BPs)^11^, are able to alter bone remodeling, interfering with tooth movement.

Zoledronic acid (ZA) is a third generation bisphosphonate, reported to have the greatest suppressive effect on bone resorption, with more intense fixation on bone, particularly on hydroxyapatites^12,13^. It is indicated for severe osteoporosis, Paget's disease, multiple myeloma and bone metastasis^14^ due to its anti-tumor^12^ and antiangiogenic^15^ effects, in addition to its effect on osteoclastogenesis^12^.

The literature has described adverse effects of orthodontic treatment associated with bisphosphonates, since osteoclastic activity is a substantial requirement for bone remodeling and, therefore, for tooth movement. Interference with tooth movement, difficulty in closing dental spaces after tooth extractions for orthodontic purposes in addition to the risk of osteonecrosis^11,16–18^ are the possible damages of the chronic use of this drug.

Bisphosphonates are preferentially incorporated into sites of active bone remodeling, increasing its concentration in the trabecular bone by two to three times, where bone tunover occurs significantly^19^. Accordingly, orthodontic tooth movement causes greater alveolar bone turnover and might further increase the uptake of bisphosphonates locally^20^, with increased risk of osteonecrosis.

In the last years, the number of adult orthodontic patients has increased dramatically, as has the use of biphosphonates either in the form of anti-osteoporosis medications or as part of a chemotherapy regimen^18^. It is important to note that the experimental model used in the present study, as well as the ZA concentrations evaluated, time and route of administration, simulate the clinical conditions of patients who have received high doses of ZA for bone metabolic disorders, such as those related to bone metastasis, and undergoes orthodontic treatment. The aging of the population increasingly exposes orthodontists to care of this group of patients, therefore, studies on the potential impact of this drug on induced tooth movement are crucial. The aim of this study was to experimentally investigate the combination of orthodontic treatment and ZA medication and to evaluate their effects on the root, periodontal ligament and alveolar bone by optical and scanning electron microscopy and computerized microtomography.

## Materials and methods

### Animals

All experimental protocols were approved by the Federal University of Ceará Committee on the Ethical Treatment of Research Animals (CEUA. No. 95/15) and performed in accordance with the ARRIVE ethical guidelines.

In order to determine the sample size, it was performed a power calculation. The sample size was determined to provide 80% power to recognize a significant difference of 20% among groups and the standard deviation of 15% with a 95% confidence interval (p = 0.05). Based on this study, a sample size of 6 rats per group was required. The animals were kept in cages with temperature-controlled rooms, with food and water ad libitum throughout the experiment.

After two weeks of acclimation to the laboratory enviroment, seventy-two male Wistar rats, weighing 200–250 g (mean age of 10 weeks), were divided, in a blind manner, into 2 groups treated with Zoledronic Acid, diluted with 0.9%w/v sodium chloride solution, following the manufacturer’s instructions, (CRISTÁLIA, Itapira, SP, Brazil) at 0.2 mg/kg ZA(0.2) or 1.0 mg /kg ZA(1.0) concentrations^16^. The vehicle control group was submitted to experimental orthodontic tooth movement and received weekly intravenous administrations of 0.9%w/v sodium chloride solution (0.1 ml/kg; Saline). The control naive group was not subjected to any procedure (Naive).

**Figure 1:**
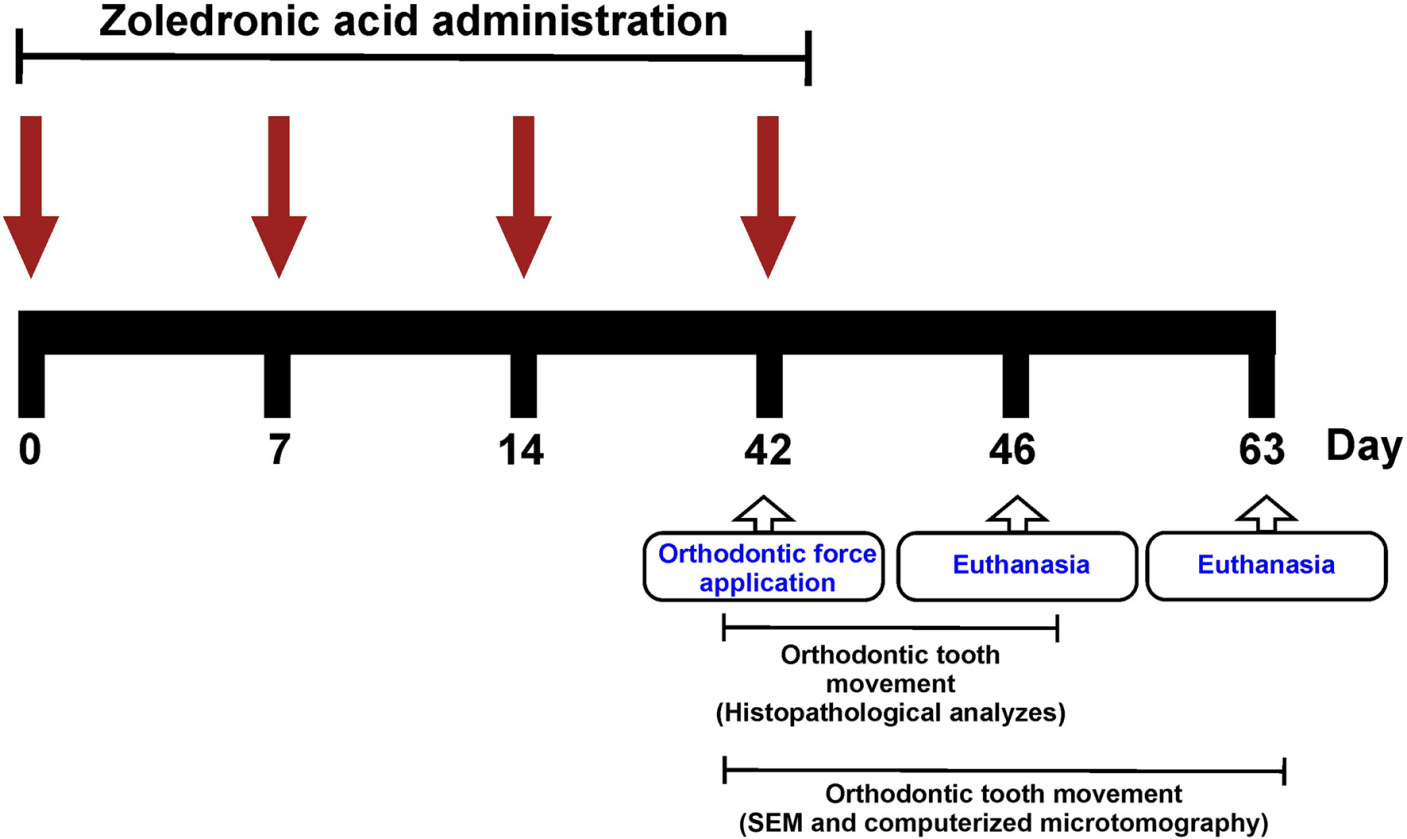
Schematic diagram illustrating the experimental design.

The animals were subjected to i.v (dorsal penile vein) administrations of ZA or saline solution, under anestesia with ketamine (60 mg/kg; i.p) and xylazine (5 mg/kg; i.p), on days 0, 7, 14 and 42. The last administration was performed immediately after the installation of an orthodontic tooth movement device. On day 46, 24 animals were euthanized (n = 6/group) and had their maxillae collected and processed for histopathological analyzes. On day 63, 48 animals were euthanized (n = 12/group) and their maxillae were collected and processed for scanning electron microscopy (SEM) (n = 6/group) and computerized microtomography (n = 6/group) examination (Fig. 1).

### Experimental orthodontic tooth movement (OTM)

**Figure 2:**
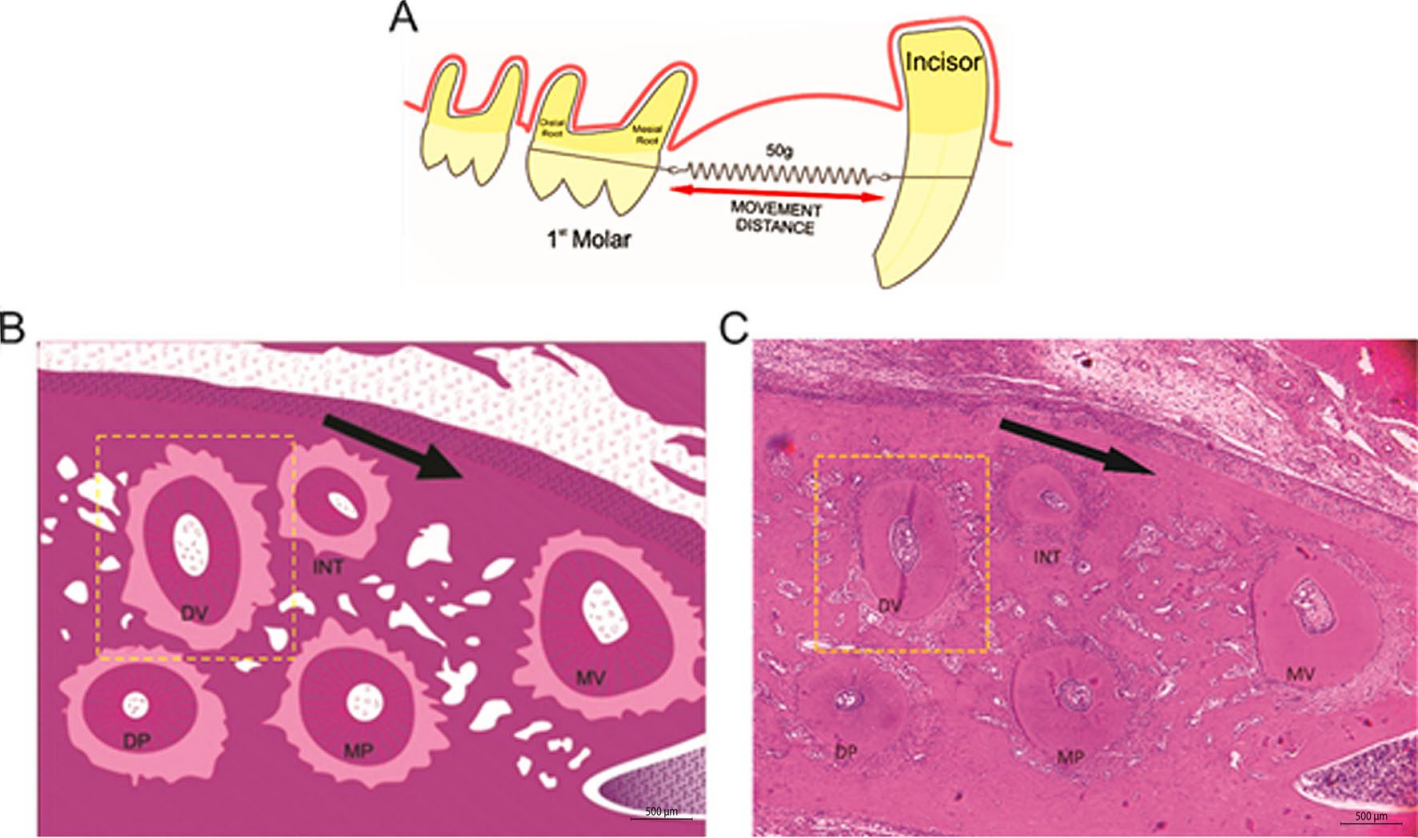
Orthodontic tooth movement (OTM) **(A)** First left upper molar surrounded by a ligature wire and Niti spring and anchored in the upper incisor teeth. **(B)** Schematic model of the roots of the rat's left upper first molar. **(C)** Photomicrograph of the roots of the rat's left upper first molar. Five roots (MV, DV, INT, MP and DP) of the maxillary first molar; cross section. Dashed line highlights the DV root, chosen for histomorphometric evaluations. The arrow indicates the direction of the applied force. Bar indicates 500 µm, HE, ×40.

For the induction of experimental orthodontic tooth movement (OTM) it was used nickel-titanium (NiTi) closed-coil springs and a 0.25-mm (MORELLI, Brazil) thick stainless steel tying wire to move the maxillary left molars mesially^7,9,11^. The animals were previously anesthetized with ketamine (70 mg/kg administered i.p., 10% Quetamina, VETNIL, São Paulo, SP, Brazil) and xylazine (10 mg/kg administered i.p., 2% CALMIUM, São Paulo, SP, Brazil). All groups, except the naïve group, were submitted to NiTi springs installation in the first left maxillary molar (force of 50gf; Fig. 2A)^9^ allowing OTM for a period of 4 or 21 days. The other end was attached to the maxillary left incisor using a tying wire and composite resin (COLTENE, Brazil). The force produced by the spring was measured and standardized using a tensiometer (MORELLI, Brazil).

### Measurement of the amount of tooth movement

The amount of tooth movement (ΔTM) was measured using a digital calliper (DIGIMESS, Brazil), positioned in the most cervical point of the molar and incisor^21^, prior to installation of coin spring and immediately following euthanasia, on day 63. The distance between the first upper left molar and the upper central incisor on the same side, measured prior to placement of the orthodontic appliance (initial measurent [im]) and after euthanasia (final measurement [fm]) were obtained. The amount of tooth movement was calculated using the following formula: ΔTM (%) = (im/fm − 1) × 100.

### Histological analysis

Four days after the installation of the springs, 24 animals (n = 6/group) were euthanized by an overdose of ketamine (300 mg/kg) and zylazine (30 mg/kg). The maxillae were removed, fixed in 10% neutral buffered formalin for 24 h, and then demineralized in 10% EDTA for 60 days, as previously described^7^. When decalcification was completed, the tissues were well rinsed in buffer and dehydrated in a series of increasing concentrations of alcohol solutions: 70% (2 changes, 30 min each), 80% (1 h), 90% (1 h) and absolute alcohol (2 changes, 1 h 30 min each). The specimens were then cleared with xylene (2 changes, 1 h 30 min each) and impregnated in 2 changes of molten paraffin wax (at 60 °C) of 1 h each and were then embedded in paraffin. Three consecutive 4 μm-thick cross sections of the left upper molar, in the mesiodistal direction, parallel to the long axis of the teeth (one for hematoxylin and eosin staining, one for Mallory staining and one for Picrosirius red staining) were consecutively cut, starting at the cervical third in the apical direction, showing the mesiobuccal and distobuccal roots^11^.

The histological stainings were performed following the manufacture’s instructions: hematoxylin and eosin (Dinâmica Química, Indaiatuba, SP, Brazil), Mallory (Bio-Optica Milano S.p.A, Milano, Italy) and Picrosirius red (Scytek, Logan, UT, USA). The distobuccal root was used to identify the periodontal ligament (PDL) areas undergoing tension and compression forces (Fig. 2B,C). The slides were examined under light microscope to evaluate the periodontal ligament and root surface in the tension and compression sides^7^. For this, 10 fields/slide (n = 6/group) were randomly photographed, under a final magnification of 200 ×, as previously described^7^.

At the tension side, blood vessels and collagen fibers were analyzed. The number of blood vessels was determined from the acquisition of images (400×) over a select area of interest (areas of intense vasculature, measuring 6 × 10^4^ μm^2^), from Mallory's trichrome stained slides (n = 6/group)^15^. The percentage of the area occupied by these vessels in relation to the total area (6 × 10^4^ μm^2^) were also evaluated using the IMAGE J 1.51 j8 software (NIH, Bethesda, MD, USA). Data were expressed as number of vessels and (%) vessel area, respectively^15^.

Anaysis of collagen fibers in PDL (at the tension side) of the first maxillary left molars were performed in slices stained with Picrosirius Red (n = 6/group) to identify the presence and type of fibrilar collagen under polarized light^22^. The data was expressed as the mean percentage ± E.P.M. of collagen content per group.

The presence or absence of hyaline areas in PDL and root resorption were identified in the compression side in slices stained with hematoxylin and eosin. The hyaline areas were delimited and measured using IMAGE J 1.51 j8 software (NIH, Bethesda, MD, USA). The data is presented as percentage of hyaline areas in relation to the total PDL area, as previouls described^21^.

In order to evaluate the resorption of root dental tissue, a line was drawn through the center of the DV root pulp using IMAGEJ 1.51 j8 software (NIH, Bethesda, MD, USA), separating the tension and compression sides. The compression side was then divided into 6 equal parts, each part corresponding to an angle of 30° (whose vertex lies at the center in the pulp). The root resorption was evaluated according to scores, based on the number of sextants that present foci of root resorption.

### Histomorphometric analysis of alveolar bone

Osteocytes and osteoclasts (cells containing more than 3 nuclei) along the margins of the DV roots were counted in 10 fields/slide (200 × magnification) using IMAGE J 1.51 j8software (NIH, Bethesda, MD, USA)^23^.

The alveolar bone resorption on the compression side was evaluated through the preservation of alveolar bone collagen from images captured from the slices stained with the HE under a confocal microscope (at 100 × magnification) (LSM 710, ZEISS, Jena, Thuringia, Germany) using the manufacturer's software (ZEN 2.1 LITE BLACK, 64-bit version, 758 MB, ZEISS, Jena, Thuringia, Germany). The excitation wavelength of collagen was taken at 488 nm (FITC-green fluorescence emission channel)^24^. The images were processed in the IMAGE J 1.51 j8 software (NIH, Bethesda, MD, USA) to calculate the percentage of resorbed area.

### Micro CT analysis

After removing the metallic ligatures, hemiarcates were collected 21 days after the installation of orthodontic appliances, dissected and stored in a − 20° C freezer. These samples were scanned using the Skyscan 1172 device (Bruker Micro CT, Kontich, Belgium) with a resolution of 7.04 μm—with the aid of an X-ray emitting device and a 100 kV voltage source, 100 μA amperage, in 180° rotation, 0.4° rotation step and 0.5 mm aluminum filter. The original images were reconstructed and converted to Bitmap images using the nRecon software (version 1.5.23) with a Smothing of 2, Ring artefact of 5 and Beam Hardening of 20%. The images were aligned with the Data Viewer morphometric visualization software (BRUKER MICRO CT, Version 1.15.4.0, Kontich, Belgium).

To evaluate the alveolar bone, a rectangle (3 mm thick × 0.5 mm height) was drawn in order to determine the limits of the evaluation. The rectangle was positioned parallel to the periodontal ligament and adjacent to the frontal wall of the alveolar bone, in order to measure the structural changes in the alveolar bone^9^. The following data were obtained on the tension and compression sides: fraction of bone volume and total volume (BV/TV), trabecular thickness (Tb.Th), number of trabeculae (Tb.N) and separation between trabeculae (Tb.Sp). These data were quantified using the CT Analyzer software (Bruker Micro CT, Version 1.15.4.0, Kontich, Belgium).

### Scanning electron microscopy (SEM) of evaluation of the roots

After euthanasia, 21 days after the installation of orthodontic appliances, the first upper left molars were removed and fixed in Karnovisky for at least 6 h, then kept in a Cacodylate buffer. All molars were washed in 1% sodium hypochlorite^9^. The tooth was placed in an eppendorf tube and left in the desiccator drying for 24 h. The fragments were assembled into stubs for metallization with gold powder (Quorum Metallist QT150ES, Quorum Technologies, Laughton, England) for scanning electron microscopy (MEV Inspect-50, FEI, Hillsboro, Oregon, USA)^25^. The root resorption gaps on mesiovestibular roots were qualitatively evaluated from images obtained at 600 × magnification^2^.

### Statical analysis

Values are given as means, and error bars are standard deviations. Normality was evaluated by Shapiro–Wilk test. Outliers were removed. The Student’s t-test or one-way ANOVA with Tukey / Games Howell post-hoc test were used to compare all measurements. The homogeneity of variances was tested by *Levene's* test. Statistical analyses were performed using the GraphPad Prism 6.0 (Graphpad Prism SOFTWARE, La Jolla, CA, USA) and SPSS 20.0 (SPSS Inc., Chicago, IL, USA) computer software programs. Probability level (p-value) < 0.05 was assumed.

### Ethical approval

All applicable international, national, and institutional guidelines for the care and use of animals were followed.

## Results

### Effects of ZA on tooth movement

**Figure 3:**
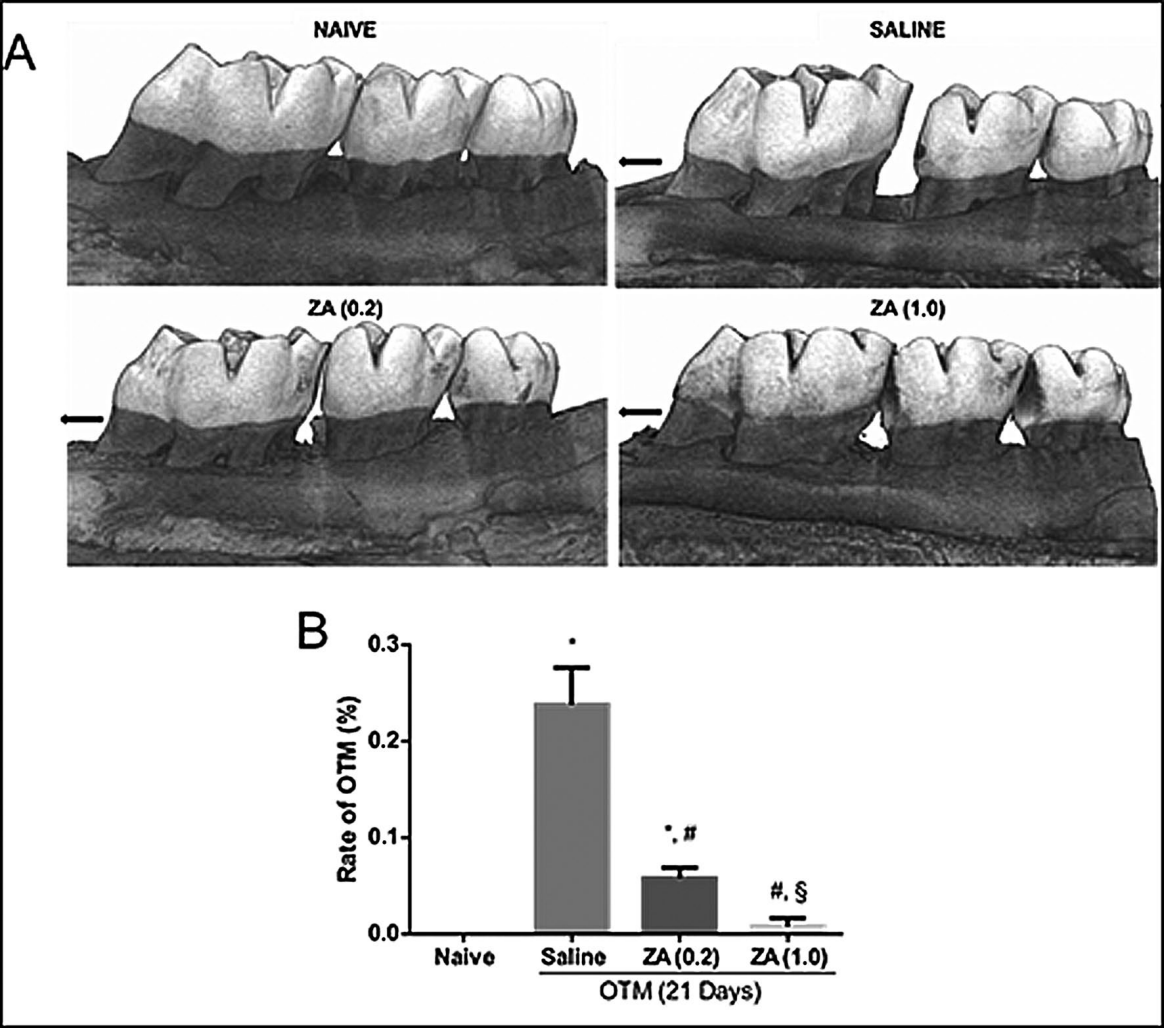
Effect of zoledronic acid on OTM with 21 days of tooth displacement in the experimental groups. **(A)** Macroscopic analysis by computed microtomography. The black arrow indicates the direction of the force on the compression side. **(B)** Bars represent mean ± SEM of 6 animals per group. (*) indicates difference when compared to the naive group (#) indicates difference when compared to the saline group. (§) indicates difference when compared to the ZA group (0.2) (ANOVA p < 0.001, Levene p = 0.001 and Games—Howell pos-hoc test).

It was observed a greater tooth displacement in the saline group when compared to the naive group, which showed null movement (p = 0.001; 95%CI = 0.16–0.30). Treatment with zoledronic acid, in both concentrations, significantly (ZA(0,2) versus Saline, p = 0.001, 95%CI = 0.11–0.24; ZA(1,0) versus Saline, p = 0.001, 95%CI = 0.16–0.29) reduced tooth displacement induced by the orthodontic appliance, as shown in Fig. 3.

### Effect of ZA on periodontal ligament

**Figure 4:**
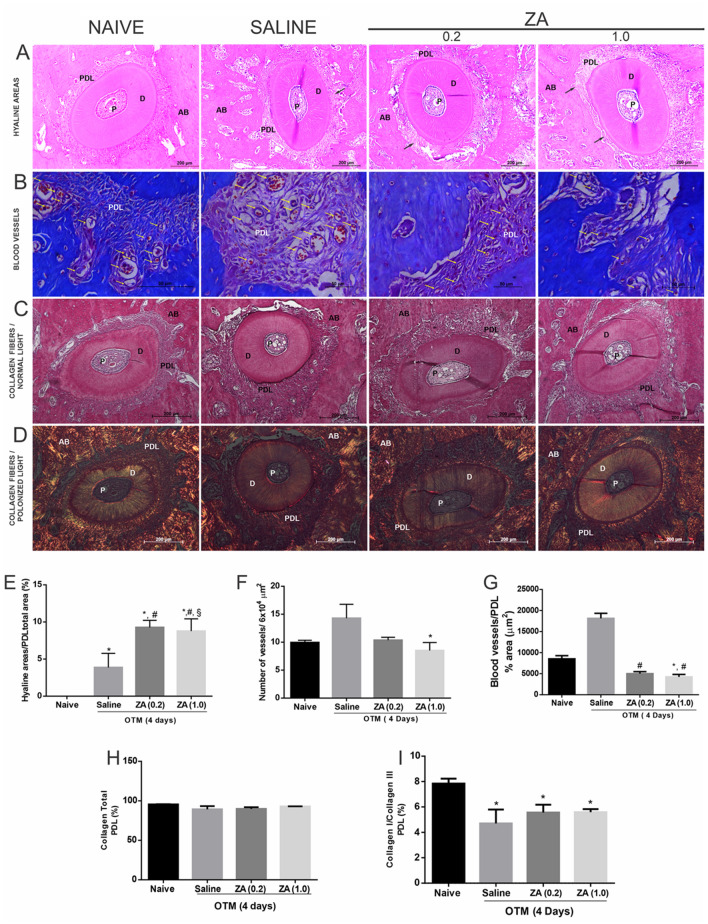
Effect of zoledronic acid on the periodontal ligament in the experimental groups Naive, Saline, ZA (0.2) and ZA (1.0). **(A)** Cross section of the root DV in HE staining **(B)** Mallory staining,  × 400 magnitude, the bars indicate 50 µm **(C)** staining with HE and **(D)** picrosirius under polarized light. *AB* bone alevolar, *D* dentin, *PDL *periodontal ligament, *P* pulp. **(E)** Histomorphometry of the periodontal ligament area with analysis in relation to the total area of the PDL. Bars represent mean ± SEM of (n = 6/group). (*) indicates difference when compared to the Naive group. (#) indicates difference when compared to the Salina group. (§) indicates difference when compared to the ZA group (0.2) (ANOVA p < 0.001, Levene p = 0.043 and Tukey pos-hoc test). (F) Analysis of the mean by fields of the amount of blood vessels. Bars represent mean ± SEM of 6 animals per group. (*) indicates difference when compared to the Salina Group (kruskal Wallis test p = 0.015 and Dunn’s pos-hoc test). (G) Analysis of the blood vessel area on the tension side of the PDL in the experimental groups. Bars represent mean ± SEM of 6 animals per group. (*) indicates difference when compared to the Naive Group and (#) indicates difference when compared to the saline group (kruskal Wallis test p < 0.001 and Dunn’s pos-hoc test). (H, I) Histopathological analysis of the type I / type III collagen fibers ratio. Kruskal–Wallis test followed by Dunn’s post-test.

The groups submitted to orthodontic force showed a significant increase (p < 0.05) in the percentage of hyaline areas in the compression side when compared to the naive group. The hyaline areas were most observed in the ZA experimental groups compared to the saline group (ZA(0,2) versus Saline, p = 0.005, 95%CI = 2.1–8.70; ZA(1,0) versus Saline, p = 0.011, 95%CI = 1.27–8.51) (Fig. 4A,E).

No statistically significant difference in the number of vessels on the tension side was observed between naive and saline groups. The ZA(1.0), but not ZA(0.2), showed reduced vascularization of periodontal tissues when compared to the saline group (p = 0.010; 95%CI = 6.72–10.28) (Fig. 4B,F). Figure 4G shows that ZA treatment reduced the total cross-sectional area of blood vessels when compared to the saline group (ZA(0,2) versus Saline, p < 0.001, 95%CI = 3.91–6.09; ZA(1,0) versus Saline, p < 0.001, 95%CI = 2.89–5.56).

There were no statistically significant differences (p > 0.05) in the percentage of collagen fibers of the periodontal ligament among the groups, but a reduction was observed (p < 0.05) in the type I/type III collagen ratio in the groups submitted to orthodontic movement when compared to the naive group (Fig. 4C,D,H,I).

### Effect of ZA on alveolar bone

**Figure 5:**
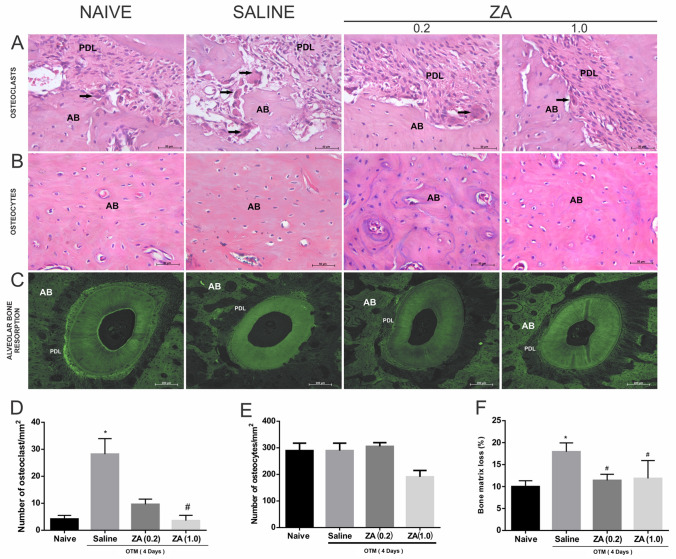
Analysis of the effect of ZA on alveolar bone in the Naive, Saline, ZA (0.2) and ZA (1.0) experimental groups. **(A)** Osteoclastic cells in the bone matrix of the alveolar bone in the DV root. *AB *alveolar bone, *PDL* periodontal ligament. The black arrow indicates osteoclastic cells. **(B)** Osteocytes in the alveolar bone matrix. **(A,B)** The bars indicate 50 µm, HE staining, × 400 magnitude. **(C)** Histopathological analysis of the resorption of the alveolar bone on the compression side by confocal microscopy of the root DV, × 100 magnitude. **(D)** Number of osteoclastic cells in the alveolar bone. Bars represent mean ± SEM of 6 animals per group. (*) indicates difference when compared to the naive group, (#) indicates difference when compared to the saline group (Kuskal–Wallis test p = 0.001 and Dunn’s pos-hoc test). **(E)** Number of osteocyte cells in the alveolar bone. **(F)** Analysis of the levels of loss in the alveolar bone matrix. Bars represent mean ± SEM of 6 animals per group. (*) indicates difference when compared to the naive group. (#) indicates difference when compared to the saline group (ANOVA p = 0.019, Levene p = 0.103 and Tukey pos-hoc test).

A higher number of osteoclasts was found in the saline group (p = 0.008; 95%CI = 21.05–35.35) when compared to the naive. Treatment with ZA, at 1.0 mg/kg, but not at 0.2 mg/kg, significantly reduced the number of osteoclastic cells when compared to the saline group (p = 0.003; 95%CI = 1.18–6.02) (Fig. 5A,D). There was no significant difference in the number of osteocytes among the experimental groups (Fig. 5B,E).

A greater loss of bone collagen was observed in the saline group when compared to the naive group (p = 0.018; 95%CI = 1.46–14.38). Zoledronic acid significantly prevented the alveolar bone matrix resorption (Fig. 5C,F).

### Effect of ZA on root resorption

**Figure 6:**
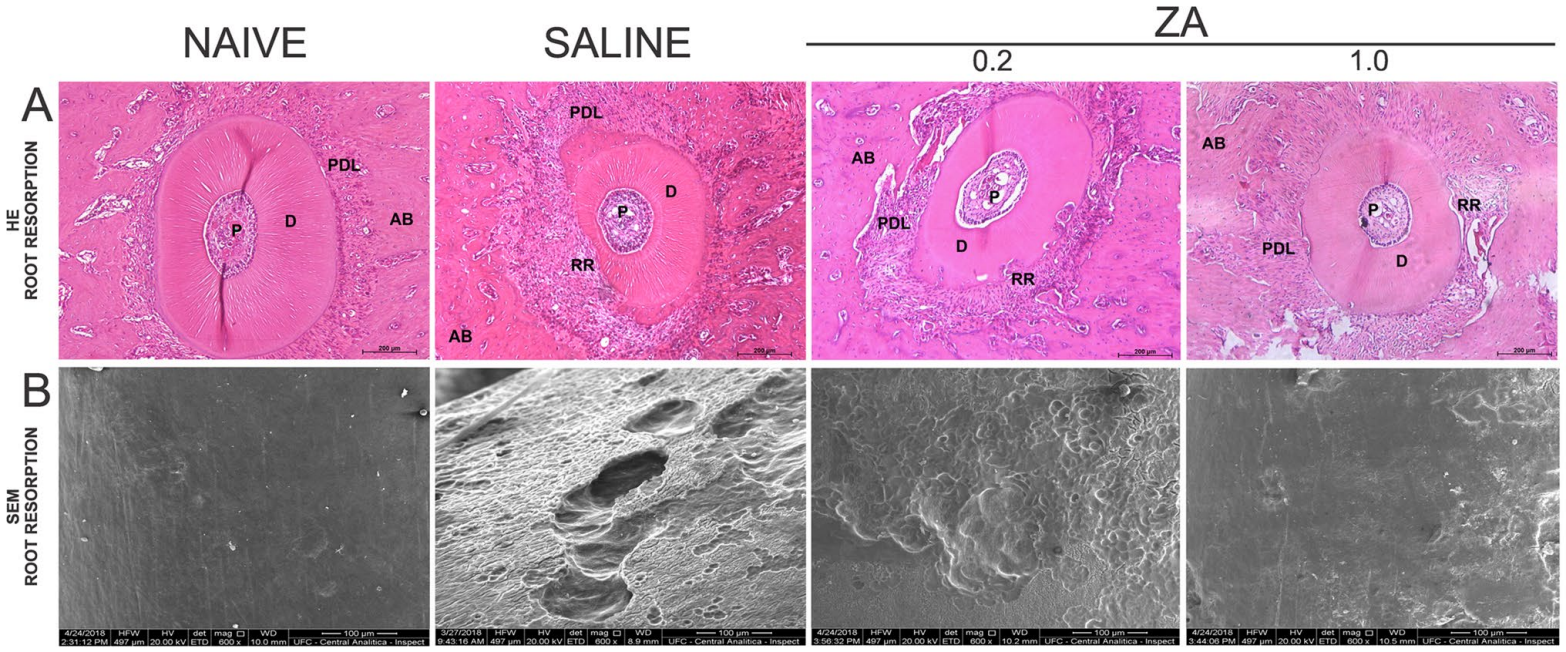
Analysis of root resorption in the Naive, Saline, ZA (0.2) and ZA (1.0) experimental groups. **(A)** Photomicrography of the distovestibular root. *RR* root resorption, *PDL* periodontal ligament, *P *pulp, *D* dentin, *AB* alveolar bone. The bars indicate 200 µm, HE staining, × 100 magnitude. **(B)** Electrophotomicrograph of the SEM analysis, the bars indicate 1 mm, × 600.

It was observed 21 days after the installation of orthodontic appliances significant differences in the microscopic analysis of tooth roots between the groups submitted to orthodontic movement and the naive group, as shown in Table 1 and Fig. 6A,B. In the naive group, the cementum and dentin exhibited intact connective tissue, with no resorption areas on the cementum surface (Fig. 6A,B). On the other hand, resorption is evident on the compression side in the saline group (Fig. 6A). SEM revelead the orthodontically induced root craters (Fig. 6B), much larger in the saline group than in the AZ treated groups. The SEM evaluation shows a normal smooth topography of the root surface of the naive group.

**Table 1:** Histopathological analysis of the effect of ZA on root resorption, expressed in scores. Significant bold value if p < 0.05.

Scores root resorption		
Days	Scores (median/maximum − mínimum)	p-value
Naive	0 (0–0)	^a, b^
Saline	6 (6–6)	^a^
ZA (0.2)	3.5 (3–4)	^b^
ZA (1.0)	2 (2–2)	^c^

(a) Association between the Naive versus Saline groups—p < 0.000. (b) Association between the Naive versus ZA groups (0.2)—p = 0.015. (c) Association between the Saline versus ZA groups (1.0)—p = 0.015 (Kruskal–Wallis test followed by pos-hoc Dunn's test).

### MicroCT analysis

**Figure 7:**
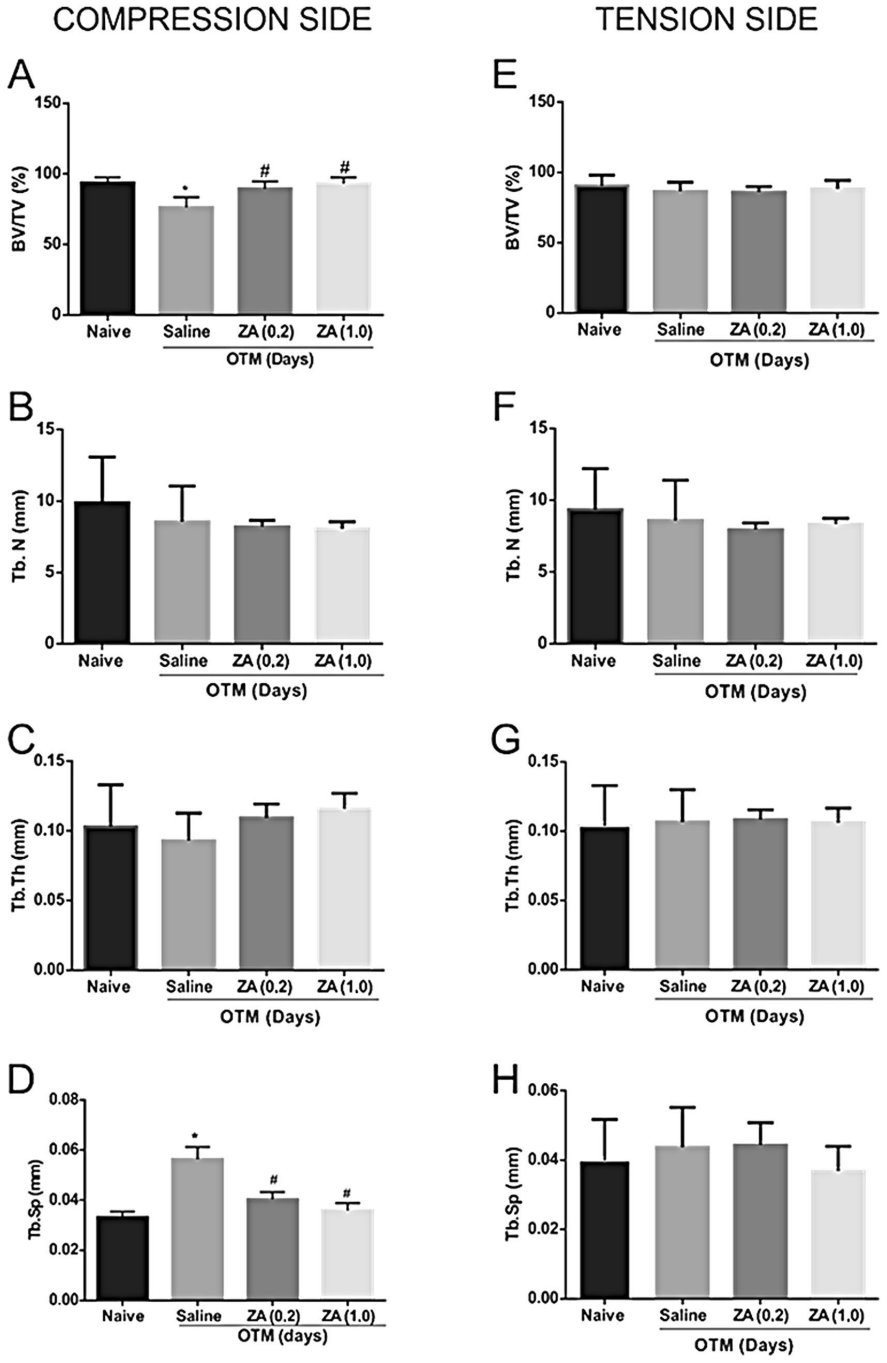
Microarchitecture analysis of trabecular bone on the compression and tension side of the DV root in the Naive, Saline, ZA (0.2) and ZA (1.0) groups. Compression side **(A)** Analysis of the inorganic portion of the bone volume fraction / total jaw bone volume (*) indicates difference when compared to the naive group (#) indicates difference when compared to the saline group. (ANOVA p < 0.001, Levene p = 0.040, Games-Howell pos-hoc test). **(B)** Measures of density of number of trabeculae (Tb.N), **(C)** thickness (Tb.Th) and **(D)** separation between trabeculae (Tb.Sp), (*) indicates difference when compared to the naive group (#) indicates difference when compared to the saline group. (ANOVA p = 0.001, Levene p = 0.250, Tukey post-hoc test). Tension side **(E)** BV/TV fraction density measurements, **(F)** number of trabeculae (Tb.N), **(G)** thickness (Tb.Th) and **(H)** separation between the trabeculae (Tb.Sp).

The induced tooth movement reduced the fraction of bone volume / total volume (BV / TV) on the compression side of the saline group when compared to the naive group (p = 0.013; 95%CI = 4.82–30.27). Treatment with ZA prevented the reduction in bone volume during OTM, characterizing a decrease in bone resorption when compared to the saline group (Fig. 7A).

An increased trabeculae separation (Tb.Sp) was observed in the saline group when compared to the naive group (p = 0.001; 95%CI = 0.01–0.036). Treatment with ZA decreased trabeculae separation when compared to the saline group (Fig. 7D). There were no significant differences in the trabeculae number (Tb.N) or in the trabeculae thickness (Tb.Th) among the groups (Fig. 7B,C).

There were no significant differences in the bone composition on the tension side among the experimental groups (Fig. 7E–H).

## Discussion

The concentrations of zoledronic acid (ZA) used in the present study were selected from previous research described in the literature that investigated whether the continuous use of bisphosphonates, including ZA, predisposes to osteonecrosis of the jaws after tooth extraction^16^. The authors used an interspecies allometric scaling for dose conversion between animals and human, based on the differences in body surface area and body weight. According to this study, the monthly administration of 4 mg of ZA, used to treat multiple myeloma in humans, corresponds to 0.60 mg/kg in Wistar rats. Based on that study, we investigated the influence of this concentration of ZA, divided into three weekly intravenously administrations of 0.20 mg/kg each, on accelerated bone metabolism induced by tooth movement and its repercussion on periodontal tissues. In addition, we also investigated the effects of a five times greater concentration, 1.00 mg/kg.

Osteonecrosis of the jaws, due to unclear mechanisms that involve changes in vascularization, is a described clinical condition that has been associated with the use of bisphosphonates, including ZA, a potent third generation bisphosphonate^26^. It has been reported that patients treated with intravenous bisphosphonate are at a higher risk compared to patients receiving oral bisphosphonate^26^. In the present study, however, despite the decrease in blood vessel area observed in the periodontal ligament of the groups treated with ZA, no macroscopic or microscopic clinical signs of necrosis were found, according to the parameters described by Ruggiero and collaborators^27^, which include fistulas and/or abscesses. On the contrary, bone integrity was verified, without necrotic areas and empty osteocytic gaps, verified by histopathological analysis.

The present study clearly showed that ZA interfered with tooth displacement, since a lower rate of orthodontic tooth movement (OTM) was found in the ZA groups compared to the untreated group. This can be explained by the decrease in osteoclasts observed in the ZA groups. It is well established the crucial role of osteoclast, as the first step for the orthodontic movement. Any interference in the function of these cells results in decreased efficiency of orthodontic treatment. In fact, ZA acts as a regulator of bone metabolism, through the inibition of osteoclastic activity, mainly by inducing apoptosis of osteoclasts and inhibiting the maturation of these cells^15–22^. Studies described in the literature reinforce our findings^21,23–28^. Different results, however, have been described by Seifi et al.^17^, who evaluated the effect of local administration (injected into the vestibule of the mesial root of the first maxillary molars) of a ZA made in Iran (called zolena) on orthodontic tooth movement and bone and root resorption in rats. Zolena did not decrease OTM but it significantly inhibited bone and root resorption and angiogenesis. The authors speculate that zolena might be less potent than its foreing counterparts.

The anti-resorptive effect of ZA treatment on alveolar bone can be seen on the compression side, where the periodontal ligament is compressed by the tooth against the alveolar bone. It is the place where the highest rate of bone resorption was observed in the untreated group, submitted to OTM (saline group). Accordinly, the literature reports that during induced-tooth movement, periodontal ligament responds to mechanical force stimulation and provides a microenvironment for cellular reactions and tissue remodeling^29^. The accumulation of M1 and M2 macrophages on the compression side of periodontal tissues has been described^30,31^. Activated M1 macrophages may secrete proinflammatory cytokines that are associated with bone loss and root resorption, while M2 macrophages release antiinflammatory cytokines. These studies suggest that the balance between M1 and M2 macrophages is the key point in root resorption and bone loss induced by orthodontic mechanical force^30,31^.

The periodontal ligament space thickness at the compression side were wider in the saline group than in AZ groups, as a consequence of the greater bone resorption observed in the untreated control group, which results in more space to be occupied by the periodontal ligament. In accordance, the number of osteoclasts was significanly higher at the compression side of the saline group compared to the group treated with the highest concentration of ZA. This finding explains the significant reduction in tooth displacement observed in the ZA groups when compared to the untreated group. The non-resorbed alveolar bone acts as a physical barrier, preventing tooth movement.

In addition to the anti-osteoclastic actions of ZA, we especulate that the smaller number of osteoclasts found in the ZA groups may be related to the lower vascularization observed in the periodontal ligament of these animals, considering that osteoclasts are formed from the fusion of monocytes that reach the bone through the bloodstream^32^. The greater compression of the periodontal ligament in the ZA group may contributes to the decrease in the number and area of blood vessels in the periodontal ligament observed in the ZA groups when compared to saline. The bone anti-resorptive effect of ZA was confirmed by confocal microscopy, as greater collagen integrity was found in the alveolar bone of ZA groups when compared to the saline group.

The computerized microtomographs (Micro-CT) analysis also confirms the anti-resorptive action of ZA in bone tissue during OTM. This analysis is extremely relevant, since the micro-CT allows a three-dimensional assessment of the alveolar bone microarchitecture^33^. In the evaluation of the trabecular bone of the saline group, it was observed that the induced tooth movement increases the separation between the trabeculae (Tb.Sp), without changing the number of these trabeculae (Tb.N) and neither the thickness of trabeculae (Tb. Th) on the compression side. Treatment with ZA maintains the number and thickness of the trabeculae, but reduces, however, the separation of these trabeculae, when compared to the saline group. This finding reflects the greater compression in the alveolar bone observed in the ZA groups compared to the saline group. The maintenance of bone matrix integrity, observed in all groups, without alteration in the microarchitecture of the alveolar bone, confirms histopathological analyzes, which did not show osteonecrosis of the alveolar bone, reinforcing that ZA did not induce bone necrosis in the present study.

In the tension side, where there is distension of the periodontal ligament, there was no significant difference between the ZA and saline groups regarding periodontal ligament space thickness. This result reinforces the fact that the decrease in tooth movement observed in the ZA group is due to the lack of bone resorption at the compression side, and not to morphological changes at the tension side, such as the lack of stretching of periodontal fibers. The confocal microscopy analyses did not show differences in the morphology and organization of the periodontal fibers at the tension side between ZA and saline groups. The micro-CT based image analyses, on the tension side, did not show differences in the microarchitecture of the alveolar bone between the saline and ZA groups, confirming that the effects of ZA on OTM are evident on the compression side.

To investigate the effects of ZA on the periodontal ligament during OTM, we evaluated the presence of hyalinic areas and also the composition and distribution of collagen fibers, the main component of the periodontal ligament, in slices stained with picrosirius red, a dye that allows visualization and differentiation of type I collagen fibers, the most abundant in the periodontal ligament, and type III^34^. This analysis was motivated by studies suggesting toxic/ inflammatory effects of ZA on periodontal ligament cells^35,36^.

The hyaline areas, described in the literature as a cell-free area, observed in the periodontal ligament of ZA groups were significantly higher when compared to the saline group, which corroborates with Kubek and collaborators^32^. This finding might be associated with greater compression of the periodontal fibers (on the compression side) observed in the ZA groups. We also found a significant decrease in vascularization in the periodontal ligament of ZA groups. Accordingly, vascular injuries have been demonstrated in the compressed areas of the periodontal ligament which undergo hyalinization of the fibrous tissue^37^.

The analysis of the periodontal ligament performed in the present study in slices stained with picrosirius red reinforce data from the literature demonstrating that during orthodontic movement, the alveolar bone and periodontal fibers are continuously remodeled and periodontal structures are dynamically reconstructed through biological reactions in the periodontal ligament^38^. In fact, no statistical differences on the collagen content between the groups submitted to OTM (saline and ZA groups) and the naive group (animals are submitted to OTM) were found, suggesting the active and continuous repair of collagen fibers during OTM.

However, under polarized light, it was clearly observed, on the traction side, a significant reduction in the type I / type III collagen ratio in the periodontal ligament of the groups submitted to orthodontic movement when compared with the naive group.

According to the literature, mature periodontal fiber collagen, type I collagen, induces metalloproteinase (MMP-1) expression in fibroblasts and osteoblasts in response to orthodontic force applied to the tooth^38^, which results in collagen degradation. During the repair of the periodontal ligament, the amount of collagen III increases considerably and is gradually replaced by mature collagen type I^39^. Our results reinforce that active and constant reconstruction of the fibers of the periodontal ligament occurs in orthodontically moved teeth. Treatment with ZA did not interfere with the repair of periodontal ligament fibers in the present work, contradicting studies that show toxic effects of ZA on periodontal ligament^35,36^.

We also evaluated, in the present work, the effects of ZA on the tooth root. This analysis was motivated by previous studies that demonstrate root resorption in experimental models of orthodontic movement. Dentin and cementum are two mineralized tissues, both with a composition similar to bone tissue, with a predominance of type I collagen. Like bone tissue, dentin and cement are degraded by clastic cells, called odontoclasts. However, the odontoclast is, morphologically and functionally, very similar to the osteoclast^28^. OTM resulted in significant root resorption and ZA prevented this effect. The inhibitory effects of ZA on osteoclasts formation and activation may explain the lower root resorption, observed in the group treated with the highest concentration of this drug. In the presente study, optical microscopy and scanning electron microscopy analyses cleary show a reduction in the number and depth of root resorption gaps in the ZA group, when compared to saline. Root resorption is an undesirable effect of orthodontic treatment, usually associated with forces of high magnitude, which promote a greater number of hyalinic (necrotic) areas and, consequently, greater formation and activation of clastic cells^17,40^. In the present study, a force of 50gf was used, based on previous studies^9,11^, which corresponds to a force that exceeds the intensity of force necessary to move a tooth with minimal tissue damage (optimal strength). In experimental tooth movement, the use of a force that exceeds the optimum force allows the occurrence of all tissue events involved in orthodontic movement in a viable time frame for experimental studies.

## Conclusion

In summary, our results contribute to a better understanding of the cellular effects of ZA and its repercussion on root, periodontal ligament and alveolar bone tissues, which is crucial for the indication of orthodontic treatment to patients who use high doses of this drug. Although in the present study the antiangiogenic effect was associated with the treatment of ZA, the bone structure was preserved with no signs of osteonecrosis.
